# The role of art therapy on quality of life of women with recent pregnancy loss: A randomized clinical trial

**DOI:** 10.1371/journal.pone.0305403

**Published:** 2024-07-25

**Authors:** Masumeh Zahmatkesh, Shahla Faal Siahkal, Fatemeh Alahverdi, Golshan Tahmasebi, Elham Ebrahimi

**Affiliations:** 1 Department of Reproductive Health Midwifery, School of Nursing & Midwifery, Tehran University of Medical Sciences, Tehran, Iran; 2 Department of Midwifery, Marand Branch, Islamic Azad University, Marand, Iran; 3 School of Nursing & Midwifery, Tehran University of Medical Sciences, Tehran, Iran; PLOS: Public Library of Science, UNITED STATES OF AMERICA

## Abstract

**Background:**

Pregnancy loss and mourning can lead to psychological adverse effects on women’s quality of life. This study aimed to evaluate the effect of art therapy on the quality of life of women with pregnancy loss.

**Methods:**

This study was a randomized clinical trial performed on 60 women who recently experienced abortion or stillbirth. After randomization in two groups (30 in each group), women in the intervention group received four session art therapy. In the control group, routine care was performed. The Perinatal Grief Scale and World Health Organization quality of life questionnaire, short version 26, was used to collect data before and eight weeks after intervention, and the result was compared before and after the intervention in both groups.

**Results:**

The mean age of participants was 26.5±4.75 years. Eight weeks after the intervention, the mean score of the total quality of life was significantly different between the two groups (348.64±13.12 vs.254.46±58.35; P>0.01). Also, all physical, psychological, social, and environmental dimensions of quality of life improved in the art therapy group compared to the control group (P>0.01).

**Conclusions:**

Art therapy could improve the quality-of-life following pregnancy loss, and can be recommended as a complementary method next to routine care.

**Trial registration:**

IRCT20200104046002N1.

## Background

Pregnancy loss such as Intra Uterine Fetal Death (IUFD), abortion, or even neonatal death can impose traumatic effects on mothers [1–3]. According to the literature, mothers start to attach to their fetuses right from the moment they find out about the pregnancy [4,5]. This level of attachment progresses during pregnancy, and mothers consider their fetus as a real person [4]. So that, grief is understandable after pregnancy loss [6]. In other words, mothers who lose their fetuses experience the same stages of grief as mothers who lose their children [7]. Grief can be complex and have an effect on multiple aspects of family life following perinatal loss [2]. Grief after pregnancy loss can become complicated if there is a mother’s psychopathology, a previous history of pregnancy loss, and social pressure to get pregnant again [8]. Complicated grief (CG) reactions can increase the risk of psychological and physical well-being [6]. Against the general perception that accepts the notion of maternal grief, severe mental symptom reports showed adverse effects which last up to 12 months or more after perinatal death [2]. There are some degrees of heterogeneity between study results related to grief’s impacts on physical and mental illnesses. In some studies, grief is considered one of the major risk factors for mental illness [1]. so, more than 30% of females who have experienced abortion also experience sorrow, depression, and anxiety; in 5% to 10% of cases, this is due to the severity and continuity of depression symptoms [9]. Nowadays, the health promotion of both mother and child, which includes improving health standards that involve all aspects of their health, is the focus of all health strategies. In this regard, quality of life has become an important issue [10]. Feizollahi et al. (2019) investigated the relationship between post-abortion grief and women’s quality of life in a descriptive-analytical study. The results of this study showed that there is a significant relationship between severe grief after abortion and low quality of life [11].

Pregnancy loss is associated with an increased risk of all-cause mortality, especially cardiovascular mortality [12] and other health problems, such as sleep quality [13] and sexual and marital health [14]. It can also influence decisions about future pregnancy [15]. The quality of life presented by WHO is considered a person’s perception of their life. It has 6 general dimensions consisting of physical, psychological, social, environmental, spiritual, and level of independence, which are to some extent similar to dimensions of complete health [16,17]. Quality of life has multiple aspects or angles which can be altered over time through changes in the personal and social values [18]. According to the best of the authors’ knowledge, the quality of life in women with pregnancy loss is lower than those without this experience [19,20]. It is accepted that post pregnancy loss grief increases the possibility of physical and psychological disorders, such as anxiety, sleep disorders, and depression in females [21,22].

One of the most effective ways to deal with the psychological problems of losing a pregnancy is to use non-pharmacological alternative methods. Educational and skillful interventions as complementary treatments can be effective in managing the adverse events of life [23]. One of the newest and most effective complementary methods is art therapy, whose effects have been shown in solving psychological problems during pregnancy [24,25]. In art therapy, art materials help people to express their feeling and become empowered to solve their internal conflicts in the presence of a trained art therapist. Moreover, there is no need for previous patients’ skills or experience to accomplish the task of art therapists [26]. As art production and reflection of it progresses, people’s awareness of themselves strengthens positive emotions, and self-confidence is created [26–28]. For this purpose, different branches of art such as using painting, sculpture, photography, collage, music, drama, poetry have been used by music therapy, drama therapy, story therapy and painting therapy [29–32].

Considering the adverse effects of grief after pregnancy failure which threatens the health of women, as well as the interest and tendency of people in society today to join alternative and non-pharmacological treatments and pay attention to the small body of research in this area, this study aimed to evaluate the effect of Art therapy (the shaped painting) on quality of life of women who were recently faced with pregnancy loss.

## Research approach and methodology

### Design of study

The present study was a randomized clinical trial that evaluated the possible impact of art therapy on women’s quality of life after pregnancy loss. This study was designed and implemented based on CONSORT 2010 guidelines. The current research was conducted on 60 women (30 in the control group and 30 in the intervention group) with abortion or stillbirth referring to the maternity wards of Baharlu and Valiasr hospitals under the supervision of the Tehran University of Medical Sciences who met the inclusion criteria. The data were collected between 21/01/2020 to 2/08/2020 and were accessed for research purposes on 30/08/2020.

### Ethics approval and consent to participate

The present study is the result of a dissertation for a master’s degree in midwifery approved by the Tehran University of Medical Sciences with an ethics code of IR.TUMS.FNM.REC.1398.102, approved by and registered in the Iranian Clinical Trial Center with a code of IRCT20200104046002N1.

#### Inclusion and exclusion criteria

The inclusion criteria were the desire to participate in the study, having a history of abortion or stillbirth in the last six weeks, having minimal literacy, and confirming bereavement using the PGS questionnaire.

The exclusion criteria were a history of mental illness or severe psychological reactions that require major interventions or referral to a psychiatrist and current use of antidepressants.

#### Sampling

After obtaining permission from the research ethics committee of the Faculty of Nursing and Midwifery of Tehran University with the ethics number IR.TUMS.FNM.REC.1398.102 and registering the research in the IRCT with the code IRCT20200104046002N1, the lead researcher attended the maternity ward of Baharlo and Valiasr Hospital. Eligible women were identified, and women who wanted to participate in the study signed the informed written consent form.

#### Randomization

Individuals were randomly assigned to the intervention and control groups. In this way, the samples that met the criteria for joining the study were first selected using the accessible sampling method. After obtaining informed consent, they completed the Prenatal Grief Scale (PGS) questionnaire. Women with active grief were included in the study. Then the subjects were randomly assigned and divided into two intervention and control groups using block randomization (block size of four). The “blockrand” package of R software was used for block randomization. Then, the obtained numbers were placed inside the secured opaque envelopes in such a way that inside each envelope one of the letters was placed in the order determined, and the envelope was closed by the staff that not involved in the research and given to the researcher. A statistical consultant did the process of randomization and concealment.

#### Intervention

In the experimental group, four sessions of art therapy were held to carry out the study by the first author who had an art therapy coaching license. In the control group routine care was received. In the intervention group, four art therapy sessions were held. Each session was 90 minutes and included the warm-up exercises, the main exercises, and the relaxation exercises.

The warm-up exercises included making a symbol with clay, recognizing contrasting colors, lines, shapes, color spots, and introducing colors.

The art therapy for groups book by Liebmann M was used to design the intervention [33]. The main exercises included paper napkin collages or pictures in the form of making a family tree, creating the current situation, a full-length picture, drawing worst fears and threatening situation, grief balloons, envelope and box, drawing a face of yourself, making a symbol of women’s talents or capabilities with a painting or collage, drawing a support circle and a relaxation table, making seasons with clay, making a bird nest, a bird and a tree hit by a storm, writing a letter to God or the partner.

The relaxation exercises included breathing, music, gratitude, listening to non-verbal music, closing the eyes, and imagining a relaxing place. At the beginning of the study, and eight weeks after that, both study groups answered the short version of the quality of life developed by WHO.

#### Outcomes

The primary outcome of this study was the assessment of the total quality of life in women with pregnancy loss, and our secondary outcomes were the physical, social, psychological, and environmental dimensions of quality of life in these women.

#### Sample size

According to SVENSK’s study [34] about the effect of art therapy on the quality of life and study power equal to 90% and alpha to 0.05, the number of sample size was estimated to be 20 people in each group, which considered 20% dropout, 25 people were selected in each group. However, we selected 30 participants for each group.


$$n=\frac{s1^{2}+s2^{2}}{(\mu 2-\mu 1)2}f(\propto ,\beta )=39$$

alpha = 0.0500

power = 0.9000

delta = 17.5000

µ1 = 67.5000  µ2 = 85.0000

sd1 = 20.3000  sd2 = 12.5700

#### Data collection tools

Data collection tools included the demographic questionnaire, the Persian version of Potvin et al.’s prenatal grief scale, and the World Health Organization’s quality of life questionnaire, short version of 26 questions (WHOQOL).

#### Demographic questionnaire

This questionnaire was a researcher-made questionnaire that included: age, level of education, occupation, Socio-economic status, number of pregnancies, number of abortions and stillbirths. The questionnaires were completed in the presence of the first author by the mothers themselves, and if necessary, explanations were given about the questions.

- *Perinatal Grief Scale Potvin et al*. *[35]*

This scale includes 33 items and three subscales including: active grief; difficult coping; and hopelessness. The scoring of the questionnaire is in the form of a 5-point Likert scale. Items No. 11 and 32 are graded in reverse. A score of 92 or higher in the total score or 34 or higher in the active grief subscale, a score of 30 or higher in the difficult coping subscale, and a score of 27 or higher in the hopelessness subscale indicate a high degree of grief. This scale shows good internal consistency with an overall alpha coefficient of 0.92 (the range of subscale correlation coefficients is from 0.86 to 0.92), and has a good stability with a range of retest correlation coefficients from 0.59 to 0.66 with a duration of 12 to 15 weeks. Also, this scale has good factorial validity, the 33-option form of PGS is correlated with the long form of PGS with a coefficient of 0.98 [36]. Siadatnezhad et al. (2018) translated this scale to Persian and assessed its validation. According to this study, the Cronbach’s alpha coefficient for the PGS total scale was 0.95, and for the PGS factors of the Persian version, it varied from 0.84 to 0.89 [37].

- *World Health Organization Quality of Life Questionnaire*, *short version 26 (WHOQOL BREF) [38]*

The World Health Organization Quality of Life Questionnaire, a short version of 26 questions, was designed in 1994 under the supervision of the World Health Organization to assess people’s perception of value and cultural systems as well as their personal goals, standards and concerns. It is international and independent of culture to evaluate people’s quality of life. The short WHOQOL consists of 26 items taken from the 100-item version of this questionnaire. This questionnaire measures 4 broad areas: physical health, psychological health, social relations and environment. In addition to these cases, this questionnaire can also evaluate public health. Questionnaire items are evaluated on a 5-item scale. A higher score indicates a better quality of life [39]. Analysis of internal consistency, item-total correlation, discriminant validity and construct validity in the results obtained from 23 countries showed that WHOQOL-BREF has good validity and reliability [40]. Rasafiani et al. (2020) translated this scale to Persian and assessed its validation. Based on the results, the content validity ratio of Persian version for all questions was between 0.7–1 and the content validity index was 0.85 [41].

#### Statistical analyses

SPSS version 18 statistical software was used for data analysis. Kolmogorov test was used to check the normality of the data. Descriptive statistics including mean, standard deviation, and relative and absolute frequency tables as well as Chi-square, Independent and Paired T-test, and Fisher’s Exact Test (one-tailed test) were performed on a significance level of p<0.05 to analyze data based on the normality of the data.

## Results

**Fig 1:**
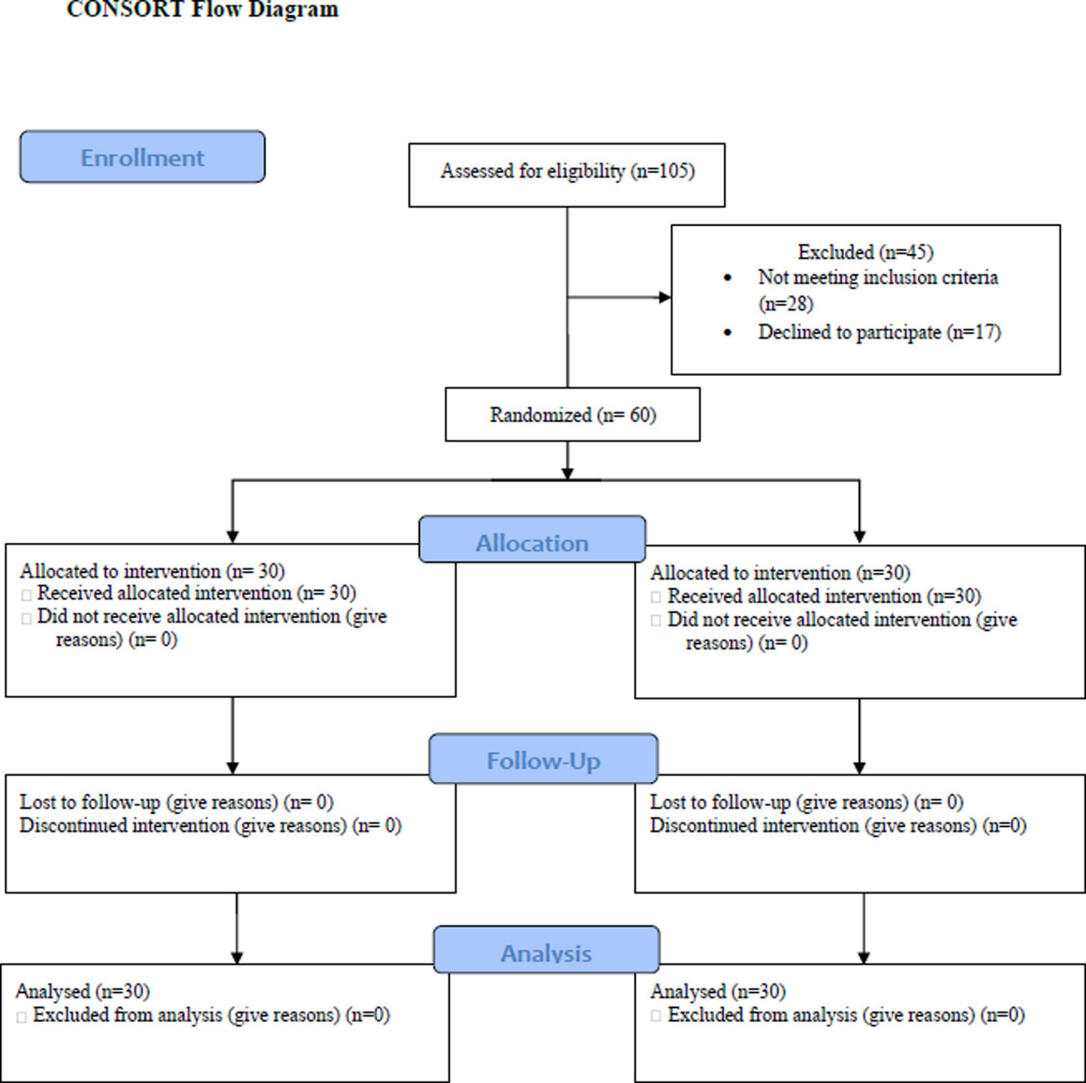
Flow of the participants through the study.

As the flow chart of the study shows, 30 people were included randomly into each group. There was no drop in the number of participants in the two groups, and finally, the analysis was done on the data obtained from 60 samples. Fig 1 represents how participants selected throughout the trial.

This study was conducted from May 2020 to the end of March 2021. The average age of women was 26.9±5.5 years in the intervention group versus 26.1±4 in the control group (P = 0.513). Table 1 shows demographic and obstetrics characteristics of participants of this study. There were no statistically significant differences in regards of demographic and obstetrics characteristics between the two groups (P>0.05).

**Table 1:** Demographic and obstetrics characteristics of participants.

Characteristics		Control (N = 30)	Intervention (N = 30)	P-value
		Mean ±SD	Mean ±SD	
		OR N (%)	OR N (%)	
**Age (y)**		26.1±4.0	26.9±5.5	0.513*
**Gestational age at the time of abortion or fetal death (week)**		26.1±1.01	26.9±0.74	0.521*
**Grief score before intervention**		43.1±4.9	44.4±4.46	0.90*
**Education**	**Under diploma**	6 (20.0%)	6 (20.0%)	0.426***
	**Diploma**	16 (53.3%)	10 (33.3%)	
	**University**	8 (26.7%)	14 (46.7%)	
**Job**	**Housewife**	18 (60.0%)	19 (63.3%)	0.282***
	**Employed**	12 (40.0%)	11 (36.7%)	
**Number of pregnancies**	**1**	15 (50.0%)	11 (36.7%)	0.415*
	**2**	3 (10.0%)	12 (40.0%)	
	**≥3**	12(40.0%)	7(23.3%)	
**Number of abortions**	**0**	2 (6.7%)	4 (13.3%)	0.856*
	**1**	19(63.3%)	17(56.7%)	
	**≥2**	9(30%)	9(30%)	
**Number of stillbirths**	**0**	26(86.7%)	21(70%)	0.577*
	**1**	4(13.3%)	6(20.0%)	
	**2**	0(0%)	3(10.0%)	
**Infertility history**	**Yes**	4(13.3%)	2(6.7%)	0.536**
	**No**	26(86.7%)	28(93.3%)	

Values are expressed as mean ± SD or N (%).

*Independent t-test

**chi-square test

*** Fisher’s Exact test (one-tailed test).

Table 2 shows the total score of quality of life before and eight weeks after intervention between the two groups. According to the results of the independent t-test, there was no difference between the two groups in the average quality of life score before the intervention (250.46±41.58 vs. 251.06±56.89; P = 0.19). However, eight weeks after the intervention, there was a statistically significant difference between the two groups, so the quality-of-life score in the intervention group increased significantly (348.64±13.12 vs.254.46±58.35; P<0.01).

**Table 2:** Comparison of the mean score of total score of quality of life after pregnancy loss in control and intervention groups, before and 8 weeks after intervention.

Group Time	Intervention		Control		T-Statistics	CI	P-value^**^
	Mean	SD	Mean	SD			
**Before intervention**	**250.46**	**41.58**	**251.06**	**56.89**	3.03	198.71–310.41	**P = 0.193**
**Eight weeks after the intervention**	**348.64**	**13.12**	**254.46**	**58.75**	4. 13	198.71–310.41	P<0.001
**P- value** ^*^	P>0.001		P = 0.37				
**T-Statistics**	4.03		3.01				
**CI**	201.38–368.78		198.71–337.56				

* Paired-T-Test

^**^ Independent t-test.

The results for the four areas of comparing the quality-of-life status are reported in Table 3. The results of the independent t-test showed that the average quality of life score before the intervention was not significantly different between the two groups in dimensions of physical health (P = 0.83), mental health (P = 0.22), social health (P = 0.53), and environmental health (P = 0.17), while after the intervention, the difference between the two groups was significant in all dimensions (P<0.01). In comparison, in the average quality of life dimensions score before and after intervention, the paired t-test showed that the average score of quality of life in the control group was not significantly different. But in the intervention group, there was a significant difference before and after eight weeks in all dimensions (P<0.01).

**Table 3:** Comparison of the mean score of dimensions of quality of life after pregnancy loss in control and intervention groups, before and 8 weeks after intervention.

Physical dimension of quality of life									
Group Time	Intervention		Control			T-Statistics	CI		P- value**
	Mean	SD	Mean	SD					
**Before intervention**	64.28	10.61	68.57	11.72		3.1	47.14–73.21		P = 0.835
**Eight weeks after the intervention**	88.33	4.18	70.23	11.49					P<0.001
P-value*	P>0.001		P = 0.06						
**T-Statistics**	4.52		5.7						
**CI**	45.6–97.13		41.23–93.13						
**Mental health dimension of quality-of-life**									
**Before intervention**	53.61	8.31	50.14	12.37		T:3.54	34.13–66.78		P = 0.22
**Eight weeks after the intervention**	87.77	5.88	53.47	14.22		5.13	75.12–96.11		P<0.001
P-value*	P>0.001		P = 0.81						
**T-Statistics**	T:4.6		T:3.14						
**CI**	CI:36.9–98.21		28.14–71.64						
**Social dimension of quality of life**									
**Before intervention**	64.44	13.29	62.77	13.79		2.34	32.87–71.41	P = 0.53	
**Eight weeks after the intervention**	88.05	9.45	61.11	13.37		3.14	49.11–112.25	P<0.001	
P-value*	P>0.001		P = 0.56						
**T-Statistics**	3.61		2.02						
**CI**	37.13–105.37		31.13–73.21						
**Environmental health dimension of quality of life**									
**Before intervention**	71.45	15.86	69.58	19.01		3.11	41.13–96.02	P = 0.17	
**Eight weeks after the intervention**	84.47	8.18	69.89	19.27		5.43	43.13–109.14	P<0.001	
**P-value** *	P>0.001		P = 0.08						
**T-Statistics**	4.15		1.03						
**CI**	56.33–118		40.79–94.13						

*Paired-T-Test

** Independent-T-Test.

## Discussions

Bereavement after the abortion or intrauterine death can lead to experience high level of the anxiety and threaten the women quality of life. In this regard and as a baseline study which forms our primary motivation, we can mention the Feizollahi’s et al study report [11]. They showed the status of Iranian women regarding the experience of postabortion grief. So that, the present study seeks to determine the probable effect of art therapy on the quality of life after pregnancy loss. The results of our study showed that the average score of quality of life in four areas, including women’s mental, physical, environmental, and social health, who had received art therapy, significantly increased compared to the control group. To the best of our knowledge, no studies have been conducted on the effect of art therapy on quality of life after abortion.

There are limited studies in the field of art therapy and quality of life in pregnancy. By examining the effect of art therapy (painting) on the sleep quality of pregnant women, Sharifpour et al. (2023), showed that art therapy is effective in increasing the sleep quality of pregnant women [42]. In another qualitative study (2023), by examining the experiences of twelve participants after performing art therapy for women with high-risk pregnancies, it was shown that art therapy helps women to express their feelings better, engage in positive distractions, create more connections, learn from others, improve optimism, and reduce anxiety [43]. In other words, art therapy has been able to influence some aspects of the quality of life, and the results of these studies are consistent with our study. Meanwhile, Wahlbeck et al. (2020) could not confirm the effect of art therapy on childbirth fear compared to midwife-oriented counseling [44].

In the field of women’s cancer, some studies have shown that art therapy can have results such as improving the quality of life in women undergoing breast cancer treatment [28,34,45]. In this regard, a study conducted by Svensk et al. considered the effect of art therapy on improving the quality of life of women affected by breast cancer in Sweden. The results of this study showed that art therapy significantly promotes all aspects of quality of life and general health of the intervention group compared to the control group [34]. Therefore, art therapy may be considered an appropriate intervention for women interested in such treatments. In line with our study result, Fancourt and Perkins reported similar positive effects of mother-infant singing (as a branch of performance art therapy [46]) on emotional closeness, anxiety, and decreasing the level of stress hormones. The result indicated that singing also has a positive impact on the acceleration of recovery from postpartum depression symptoms [47].

Studies have also shown that art therapy can improve depression and anxiety as well as the quality of life of people through mindfulness [48,49]. One of these studies is the Jang study, which was conducted in 2016 to determine the impact of mindfulness-based art therapy on breast cancer patients. The results of this study showed that art therapy could significantly reduce depression and anxiety as well as health-related quality of life in the intervention group compared to the control group [50]. In this study, which was performed on women with breast cancer, a similar goal was pursued with the aim of the present study, which is to pay attention to the pressures and psychological problems of patients, especially women, in addition to their physical problems; problems that, despite their great importance in the quality of life and even the process of recovery of patients, are usually ignored and no intervention is done to address and eliminate them [21,51,52]. The results of that study, according to the intervention similar to our study (art therapy), are in line with the present study in improving the quality of life and reducing anxiety, and indicate the applicability of this type of intervention in improving the quality of life.

The results of all the above studies showed that art therapy has a great effect on improving various psychological problems such as anxiety, and depression, and also improving the quality of life. Although, the studies were different in terms of the target group, variables, method, number and time and duration of intervention, the results show reduced levels of anxiety and depression, as well as improved quality of life. Through art therapy, people seem to learn how to accept a problem, cope with it, and believe in their ability to solve problems, and in general, art therapy increases people’s self-efficacy. Another advantage of art therapy is that due to its lack of need for artistic skills, it can be used with the least and simplest facilities available and may be used to solve various mental problems. Complementary art therapy, in addition to other interventions in other studies such as psychological grief counseling and psychological supportive interventions, has a similar effect on psychological problems following pregnancy loss, but it can be done by taking short courses.

### Limitations

The intervening variables of this research include mental and emotional state, cultural and social contexts, individual differences between patients, the complexity of the bereavement problem in different people, which could be controlled due to the random selection of people in groups. On the other hand, the questionnaires were completed in two groups through self-report, which may affect the accuracy of the data. Finally, Due to the fact that the number of stillbirths in our study was very low and most of the cases were considered abortions, a separate analysis was not performed.

## Conclusion

Bereavement after the abortion or intrauterine death can lead to experience high level of the anxiety and threaten the women quality of life. According to the study result, art therapy can be considered as a supportive care for women who experience the pregnancy loss.

## Supporting information

S1 Checklist CONSORT 2010 checklist of information to include when reporting a randomised trial*. (DOC)

S1 File (PDF)

S2 File (PDF)

## Abbreviations

IUFD: Intra Uterine Fetal Death
PGS: perinatal grief scale
CG: complicated grief
WHO: World Health Organization
WHOQOL: World Health Organization’s quality of life questionnaire
