# Correction: The role of art therapy on quality of life of women with recent pregnancy loss: A randomized clinical trial

**DOI:** 10.1371/journal.pone.0321322

**Published:** 2025-03-27

**Authors:** Masumeh Zahmatkesh, Shahla Faal Siahkal, Fatemeh Alahverdi, Golshan Tahmasebi, Elham Ebrahimi

The affiliation for the fifth author is incorrect. The correct affiliation is not indicated. Elham Ebrahimi is not affiliated with #1 but with: Nursing and Midwifery Care Research Center, Department of Reproductive health and Midwifery, Tehran University of Medical Sciences, Tehran, Iran.
