# Supplementary material for: The role of art therapy on quality of life of women with recent pregnancy loss: A randomized clinical trial
Source: PLoS One. 2024 Jul 25;19(7):e0305403. doi: 10.1371/journal.pone.0305403 (PMC11271899; doi:10.1371/journal.pone.0305403)
Supplement: S1 Checklist — (DOC) [file pone.0305403.s001.doc]

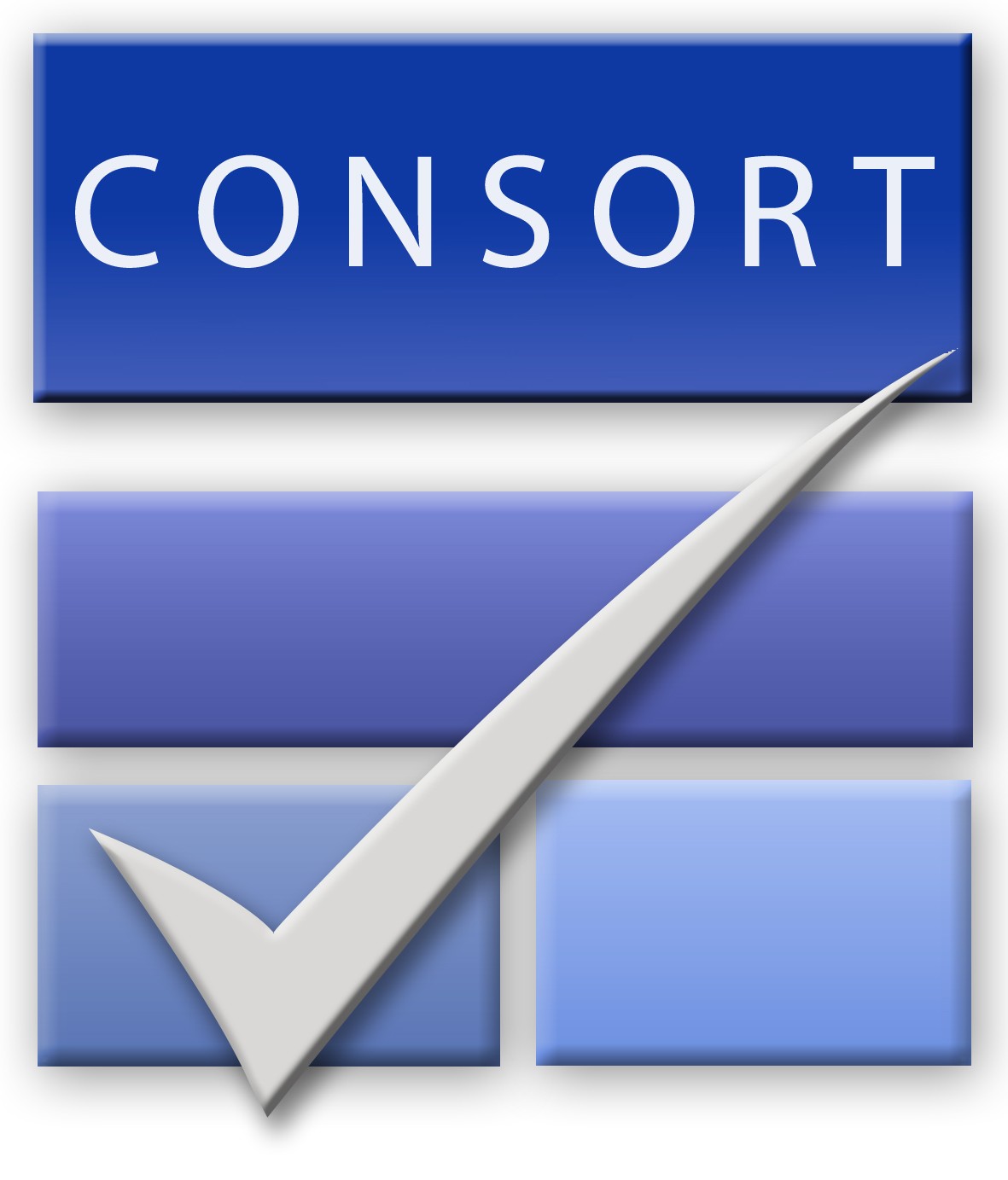
CONSORT 2010 checklist of information to include when reporting a randomised trial*

| Section/Topic | Item No | Checklist item | Reported on page No |
| --- | --- | --- | --- |
| Title and abstract | | | |
|  | 1a | Identification as a randomised trial in the title | Page 1- line 1-2 |
|  | 1b | Structured summary of trial design, methods, results, and conclusions (for specific guidance see CONSORT for abstracts) | Page 1- line 4-20 |
| Introduction | | | |
| Background and objectives | 2a | Scientific background and explanation of rationale | Page 2- line 26-68 |
|  | 2b | Specific objectives or hypotheses | Page 2- line 69-73 |
| Methods | | | |
| Trial design | 3a | Description of trial design (such as parallel, factorial) including allocation ratio | Page 3,4- line 75-82 |
|  | 3b | Important changes to methods after trial commencement (such as eligibility criteria), with reasons | Page4- line 83-88 |
| Participants | 4a | Eligibility criteria for participants | Page4- line 83-88 |
|  | 4b | Settings and locations where the data were collected | Page4- line 89-95 |
| Interventions | 5 | The interventions for each group with sufficient details to allow replication, including how and when they were actually administered | Page4,5- line 96-122 |
| Outcomes | 6a | Completely defined pre-specified primary and secondary outcome measures, including how and when they were assessed | Page5,6- line 123-126 |
|  | 6b | Any changes to trial outcomes after the trial commenced, with reasons | N/A |
| Sample size | 7a | How sample size was determined | Page6- line 127-137 |
|  | 7b | When applicable, explanation of any interim analyses and stopping guidelines | N/A |
| Randomisation: |  |  |  |
| Sequence generation | 8a | Method used to generate the random allocation sequence | Page 5- line 102-103 |
|  | 8b | Type of randomisation; details of any restriction (such as blocking and block size) | Page 5- line 102-103 |
| Allocation concealment mechanism | 9 | Mechanism used to implement the random allocation sequence (such as sequentially numbered containers), describing any steps taken to conceal the sequence until interventions were assigned | Page 5- line 102-104 |
| Implementation | 10 | Who generated the random allocation sequence, who enrolled participants, and who assigned participants to interventions | Page 5- line 104 |
| Blinding | 11a | If done, who was blinded after assignment to interventions (for example, participants, care providers, those assessing outcomes) and how | N/A |
|  | 11b | If relevant, description of the similarity of interventions | N/A |
| Statistical methods | 12a | Statistical methods used to compare groups for primary and secondary outcomes | Page 7,8- line 171-177 |
|  | 12b | Methods for additional analyses, such as subgroup analyses and adjusted analyses | Page 7,8- line 171-177 |
| Results | | | |
| Participant flow (a diagram is strongly recommended) | 13a | For each group, the numbers of participants who were randomly assigned, received intended treatment, and were analysed for the primary outcome | Page 8- line 179-181 |
|  | 13b | For each group, losses and exclusions after randomisation, together with reasons | Page 8- line 179-181 -FIG.1 |
| Recruitment | 14a | Dates defining the periods of recruitment and follow-up | Page 8- line 181-182 |
|  | 14b | Why the trial ended or was stopped | Page 8- line 181-182 |
| Baseline data | 15 | A table showing baseline demographic and clinical characteristics for each group | Page 8- line 182-186- table.1 |
| Numbers analysed | 16 | For each group, number of participants (denominator) included in each analysis and whether the analysis was by original assigned groups | Page 8- line 181-182 |
| Outcomes and estimation | 17a | For each primary and secondary outcome, results for each group, and the estimated effect size and its precision (such as 95% confidence interval) | Page 8- line 187-203 |
|  | 17b | For binary outcomes, presentation of both absolute and relative effect sizes is recommended | Page 8- line 187-203 |
| Ancillary analyses | 18 | Results of any other analyses performed, including subgroup analyses and adjusted analyses, distinguishing pre-specified from exploratory | Page 8- line 187-203 |
| Harms | 19 | All important harms or unintended effects in each group (for specific guidance see CONSORT for harms) | N/A |
| Discussion | | | |
| Limitations | 20 | Trial limitations, addressing sources of potential bias, imprecision, and, if relevant, multiplicity of analyses | Page 11- line 251-257 |
| Generalisability | 21 | Generalisability (external validity, applicability) of the trial findings | Page 11- line 251-252 |
| Interpretation | 22 | Interpretation consistent with results, balancing benefits and harms, and considering other relevant evidence | Page 9,10- line 202-250 |
| Other information | | |  |
| Registration | 23 | Registration number and name of trial registry | Page 11- line 269-270 |
| Protocol | 24 | Where the full trial protocol can be accessed, if available | Page 11- line 267-271 |
| Funding | 25 | Sources of funding and other support (such as supply of drugs), role of funders | Page 12- line 278-280 |

*We strongly recommend reading this statement in conjunction with the CONSORT 2010 Explanation and Elaboration for important clarifications on all the items. If relevant, we also recommend reading CONSORT extensions for cluster randomised trials, non-inferiority and equivalence trials, non-pharmacological treatments, herbal interventions, and pragmatic trials. Additional extensions are forthcoming: for those and for up to date references relevant to this checklist, see [www.consort-statement.org](http://www.consort-statement.org/).
