# Supplementary material for: The role of art therapy on quality of life of women with recent pregnancy loss: A randomized clinical trial
Source: PLoS One. 2024 Jul 25;19(7):e0305403. doi: 10.1371/journal.pone.0305403 (PMC11271899; doi:10.1371/journal.pone.0305403)
Supplement: S1 File — (PDF) [file pone.0305403.s002.pdf]

The effect of art therapy on anxiety and quality of life following pregnancy loss

Protocol summary

Study aim

Determining the effect of art therapy on anxiety and quality of life following pregnancy loss

Design

Clinical trial with control and intervention groups, randomized with blocking method

Sample number 60 (30 people in each group)

Settings and conduct

Individuals enter the study if they wish and have inclusion criteria. At the beginning of the referral, the prenatal grief scale of Putin et al will be completed by clients and included in the study if the grief is confirmed. Random allocation will be done by blocking method. Samples will then be informed of the subject and the informed consent form will be completed. And they complete the demographic, mourning, anxiety, and quality of life questionnaires. Samples will be divided into 4 90-minute sessions of two sessions per week of painting and pottery as planned, and the anxiety and mourning questionnaires will be completed after two sessions of art therapy and after the course is completed. Eight weeks after the intervention, all three questionnaires will be completed again. During the intervention period, no intervention will be performed in the control group except for routine medical care following pregnancy loss.

Participants/Inclusion and exclusion criteria

Inclusion criteria: 1. Having a history of miscarriage or stillbirth in the last 6 weeks 2. Minimum literacy 3. Willingness to participate in the study 4. Confirm grief by PGS questionnaire 5. Residence in Tehran Exclusion criteria: 1. Mental illness 2. Severe psychiatric reactions that require referral to psychiatry and basic interventions. 3. Current use of antidepressants 4. Unwillingness to participate in the study 5. Failure to attend at least one art therapy session

Intervention groups

Art Therapy Recipient Group Control group (not receiving art therapy)

Main outcome variables

Quality of life; anxiety.

General information

Reason for update

Acronym

IRCT registration information

IRCT registration number: **IRCT20200104046002N1**

Registration date: **2020-01-07, 1398/10/17**

Registration timing: **registered_while_recruiting**

Last update: **2020-01-07, 1398/10/17**

Expected recruitment start date

2019-10-23, 1398/08/01

Expected recruitment end date

2020-02-20, 1398/12/01

Actual recruitment start date

empty

Actual recruitment end date

empty

Trial completion date

empty

Scientific title

The effect of art therapy on anxiety and quality of life following pregnancy loss

Public title

The effect of art therapy on anxiety and quality of life following pregnancy loss

Purpose

Supportive

Inclusion/Exclusion criteria

Inclusion criteria:

Having a history of miscarriage or stillbirth in the last 6 weeks Minimum literacy Willingness to participate in the study Mourning confirmation by PGS questionnaire Residence in Tehran

Exclusion criteria:

Developing mental illness Severe psychological reactions that require referral to psychiatry and basic interventions. Current use of antidepressants Unwillingness to participate in the study Failure to attend at least one art therapy session

Age

No age limit

Gender

Female

Phase

N/A

Groups that have been masked

No information

Sample size

Target sample size: **60**

Randomization (investigator's opinion)

Randomized

Randomization description

This will be done by blocking method. For this purpose, the four blocks selected will be assigned to the intervention group letter A and the control group letter B. The 6 possible modes will be written on separate cards and assigned to each card a number (from one to six), then the numbers will be written on the paper and randomly removed. Until the expected sample number is completed. Sequence concealment will be carried out using envelopes in a package such that one of the letters is placed in the order specified and then the envelope is closed and provided to the researcher. The process of random allocation and concealment is performed by the statistical consultant.

Blinding (investigator's opinion)

Not blinded

Blinding description

Placebo

Not used

Assignment

Parallel

Other design features

Secondary Ids

empty

Ethics committees

1

Description of health condition studied

Anxiety after pregnancy loss

ICD-10 code

ICD-10 code description

2

Description of health condition studied

Mourning after losing pregnancy

ICD-10 code

ICD-10 code description

3

Description of health condition studied

Quality of life following pregnancy loss

ICD-10 code

ICD-10 code description

Primary outcomes

1

Description

Quality of Life: According to the World Health Organization Questionnaire, the short version of 26 questions indicates a score of 26 to 59 inadequate quality of life, 60 to 89 average quality of life, and a score of 90 to 130 appropriate quality of life.

Timepoint

At first, after two sessions of intervention, at the end of the intervention and 8 weeks after the intervention

Method of measurement

WHO questionnaire, short version 26 questions

2

Description

Explicit anxiety: 20 to 31 mild anxiety score, 32 to 42 moderate to low anxiety score, 43 to 53 moderates to high anxiety score, 54 to 64 moderately severe anxiety, 65 to 75 severe anxiety score, and 76 to 80 very severe anxiety score, respectively. Show.

Timepoint

At first, after two sessions of intervention, at the end of the intervention and 8 weeks after the intervention

Method of measurement

State-Trait Anxiety Inventory (STAI) questionnaire

3

Description

Hidden anxiety: 20 to 31 mild anxiety score, 32 to 42 moderate to low anxiety score, 43 to 52 moderate to high anxiety score, 53 to 62 moderately severe anxiety, 63 to 72 severe anxiety score, and 73 to 80 very severe anxiety scores, respectively. Show.

Timepoint

At first, after two sessions of intervention, at the end of the intervention and 8 weeks after the intervention

Method of measurement

State-Trait Anxiety Inventory (STAI) questionnaire

4

Description

Mourning: Earning a score of 92 or higher on an overall score of 34 or higher on the Active Mourning subscale or a score of 30 or higher on the Hard Coping subscale and a score of 27 or higher on the Despair subscale indicates a high degree of mourning.

Timepoint

At first, after two sessions of intervention, at the end of the intervention and 8 weeks after the intervention

Method of measurement

prenatal grief scale

Secondary outcomes

empty

Intervention groups

1

Description

Intervention group: Intervention group: Samples of this group will be divided into 4 sessions of 90 minutes, two sessions per week of painting and pottery, and the anxiety and mourning questionnaire will be completed after two sessions of art therapy and after the end of the course. Eight weeks after the intervention, all three questionnaires will be completed again.

Category

Rehabilitation

2

Description

Control group: Control group: Samples In this group, there will be no art therapy intervention.

Category

Rehabilitation

Recruitment centers

1

Recruitment center

Name of recruitment center

Baharloo Hospital

Full name of responsible person

Street address

Railway Square, Behdari Street

City

Tehran

Province

Tehran

Postal code

12299

Phone

+98 21 5565 8500

Email

Hosp_baharloo@tums.ac.ir

Web page address

http://medicine.tums.ac.ir/baharlou

Sponsors / Funding sources

1

Sponsor

Name of organization / entity

Tehran University of Medical Sciences

Grant code / Reference number

Is the source of funding the same sponsor organization/entity?

Yes

Title of funding source

Tehran University of Medical Sciences

Proportion provided by this source

100

Public or private sector

Public

Domestic or foreign origin

Domestic

Category of foreign source of funding

empty

Country of origin

Type of organization providing the funding

Academic

Person responsible for general inquiries

Deidentified Individual Participant Data Set (IPD)

Undecided - It is not yet known if there will be a plan to make this available

Study Protocol

Undecided - It is not yet known if there will be a plan to make this available

Statistical Analysis Plan

Undecided - It is not yet known if there will be a plan to make this available

Informed Consent Form

Undecided - It is not yet known if there will be a plan to make this available

Clinical Study Report

Undecided - It is not yet known if there will be a plan to make this available

Analytic Code

Undecided - It is not yet known if there will be a plan to make this available

Data Dictionary

Undecided - It is not yet known if there will be a plan to make this available
