# Supplementary material for: The role of art therapy on quality of life of women with recent pregnancy loss: A randomized clinical trial
Source: PLoS One. 2024 Jul 25;19(7):e0305403. doi: 10.1371/journal.pone.0305403 (PMC11271899; doi:10.1371/journal.pone.0305403)
Supplement: S2 File — (PDF) [file pone.0305403.s003.pdf]

Title: The Impact of Art Therapy on Anxiety and Quality of Life Following Pregnancy Loss
Tracking Code: 44008
Researcher:
Project Code: 961137008
Ethics Code:
Initial Registration Date: 1398/03/04
Submission Date:
Last Edit Date:
Primary Target Center: School of Nursing and Midwifery
Secondary Target Center:
Tertiary Target Center:

General Information and Abstract of Thesis

Title in English:
The effect of art therapy on anxiety and quality of life following pregnancy loss

Keywords:
art therapy, pregnancy loss, quality of life, anxiety, prenatal grief, PGS

Summary of the necessity of conducting the thesis:

Pregnancy, childbirth, and motherhood are among the most significant events in women's lives and represent the pinnacle of the divine art of creation. The pregnancy period and beyond are vital parts of a family's existence and hold a special place in the social life of a community; the physiological transition from pregnancy to motherhood entails significant physical and psychological changes in a woman. Any deviation from the natural course of these changes, both physically and mentally, can create problems for the mother and subsequently for the family. For example, failure to complete the pregnancy process, including miscarriage and stillbirth, can lead to feelings of loss and grief in women. Grieving fundamentally helps individuals cope with the impact of both major and minor losses in life. Failing to navigate the grieving process within its natural framework may

result in psychological and even physical issues in women, such as various mental disorders including depression, anxiety attacks, suicidal thoughts, sexual dysfunction, sleep disorders, decreased self-esteem, eating disorders, difficulties in communicating with others, and ultimately a change in the quality of life. One of the most effective ways to combat anxiety and depression is through non-pharmacological interventions. Studies have shown that educational and skill-based interventions are effective in reducing depression and can improve the quality of life for mothers. Recently, the use of complementary therapies for depression treatment has been under investigation. One of the newer and more effective complementary therapies is art therapy, the benefits of which in solving psychological problems have been confirmed. Given that the duty of fertility health service providers is to consider all aspects of fertility health, its process and function, it seems that grief and its consequences have not received enough attention in follow-up care, and these types of care are lacking in the assessment of health service needs both from the perspective of providers and patients. On the other hand, considering that the empowerment of women in self-care has been a focus in recent years in international documents, facilitating the psychological adaptation process for women is also one of the important goals of midwifery care. Healthcare professionals play a vital role in creating positive changes in parents and preparing them for pregnancy and healthy parenting in the future. Therefore, given the burden of grief after pregnancy loss, the possibility of its progression towards depression, the current interest in alternative and non-pharmacological therapies, and the limited studies in this area, the necessity of conducting such an important and practical investigation is undeniable.

Summary of Implementation Method and Analysis Approaches:

This research is a parallel randomized intervention study examining the correlation between art therapy, quality of life, and anxiety in women following pregnancy loss in hospitals affiliated with Tehran University of Medical Sciences. The research environment includes hospitals affiliated with Tehran University of Medical Sciences. The research population includes all women who have experienced miscarriage and stillbirth. The research sample, approved by the statistician with a sample size of 60 participants from

the research population, will be divided into intervention and control groups using block randomization.

Inclusion Criteria: History of miscarriage or stillbirth within the past 6 weeks, at least basic literacy skills, willingness to participate in the study, and grief confirmation using the Perinatal Grief Scale before childbirth questionnaire.

Exclusion Criteria: Mental illnesses, severe psychological reactions requiring referral to psychiatry and essential interventions, current use of antidepressant medication, unwillingness to participate in the study, and absence from all art therapy sessions.

Data collection tools in this research include:

1. Demographic information questionnaire including: age, education level, number of pregnancies, number of childbirths, number of miscarriages.
2. Perinatal Grief Scale (PGS) consisting of 33 items and three subscales: active grief (11 questions), difficulty coping (11 questions), and despair (11 questions), designed to measure grief in the loss before childbirth (miscarriage, ectopic pregnancy, fetal death, and neonatal death). The questionnaire scoring is on a 5-point Likert scale with corresponding scores of 1, 2, 3, 4, and 5 for options "strongly disagree," "disagree," "neither agree nor disagree," "agree," and "strongly agree" respectively.
3. World Health Organization Quality of Life questionnaire, short version with 26 items designed to assess quality of life in four dimensions: health, psychological health, social relationships, and environmental health.
4. State-Trait Anxiety Inventory - A self-report psychometric scale measuring two distinct but related concepts of anxiety. Situational anxiety refers to consciously perceived emotional situations causing tension, fear, restlessness, worry, and increased nervous system activity. Personal trait anxiety is individual differences in the disposition to perceive or evaluate stressful situations as threatening or dangerous. Responses to these situations lead to increased anxiety (Lotfi Afshar, 1997).

Initially, women will complete the grief questionnaire, and if individual grief is confirmed, they will be included in the study. The intervention group will receive two sessions per

week, totaling four 90-minute art therapy sessions following a designed protocol. The participants will complete the anxiety, grief, and quality of life questionnaires are completed by participants at the beginning of the study. After 2 weeks of intervention and at the end of the intervention, the grief and anxiety questionnaires are completed again. Eight weeks after the intervention, all three questionnaires will be completed by participants. Upon entry into the study, participants will respond to the quality-of-life questionnaire. They will also respond to the quality-of-life questionnaire with a 4-week interval from the end of the intervention, both before and after the intervention, with a 2-week interval between assessments. The scores before and after the intervention will be compared using paired t-test.

No interventions will be made for the control group during the study period. However, after this time and for ethical considerations, if participants in the control group desire, a more condensed session (2 sessions) with the same content will be held for the control group.

Registration General, Specific, and Practical Objectives Main Objectives of the Plan:

- Determining the effect of art therapy on anxiety and quality of life following pregnancy loss in women attending hospitals affiliated with Tehran University of Medical Sciences in 2019.

Subsidiary Objectives of the Plan:

- Comparing the average grief score following pregnancy loss in the intervention and control groups at the beginning, after two intervention sessions, at the end of the intervention, and 8 weeks after the completion of the intervention in women attending hospitals affiliated with Tehran University of Medical Sciences in 2019.
- Comparing the average clear anxiety score following pregnancy loss in the intervention and control groups at the beginning, after two intervention sessions, at the end of the intervention, and 8 weeks after the completion of the intervention in women attending hospitals affiliated with Tehran University of Medical Sciences in 2019.

- Comparing the average hidden anxiety score following pregnancy loss in the intervention and control groups at the beginning, after two intervention sessions, at the end of the intervention, and 8 weeks after the completion of the intervention in women attending hospitals affiliated with Tehran University of Medical Sciences in 2019.
- Comparing the quality-of-life score following pregnancy loss in the intervention and control groups before and 8 weeks after the intervention in women attending hospitals affiliated with Tehran University of Medical Sciences in 2019.

Practical Objectives of the Plan: Considering that the responsibility of fertility healthcare providers is to pay attention to all aspects of fertility health, it seems that there has not been enough attention to grief issues and the consequences of this phenomenon in the follow-up services, as indicated by both healthcare providers and patients in healthcare service needs assessment. If the results of this study demonstrate the impact of art therapy on various aspects of grief, the improvement of the quality of life and anxiety of these women, it can be deemed cost-effective and free of any side effects. In addition, it can empower women in self-care, making it a beneficial method for controlling pre-birth grief.

Introduction - Statement of the Problem

Pregnancy, childbirth, and motherhood are among the most important events in a woman's life and represent the pinnacle of creative artistry and manifestations of divine power (1,2). The period of pregnancy and beyond is a crucial part of family existence and holds a special place in the social life of a community (3) the physiological transition from pregnancy to motherhood signifies a profound physical and psychological change in a woman. This change impacts all bodily systems and can deeply influence women's emotions, behaviors, and thoughts, leading to heightened emotional sensitivity towards psychological stimuli, changes in feelings and attitudes towards children, and better adaptation to the maternal role, affecting future pregnancies and childbirth as well. (4-7)

Any deviation from the natural course of these changes, whether physical or psychological, can create problems for the mother and subsequently for the family. (8) failure to complete the pregnancy process, including miscarriage and stillbirth, can evoke

feelings of failure and grief in women. Grief and mourning, like death and other fundamental issues, are realities of life. An issue that humans consistently face, and sometimes this confrontation occurs at a young age due to pregnancy loss. (8,9) The natural stages of grief include shock and denial, increased awareness (feelings: grief, guilt, anger. Searching, bargaining), perception of the issue (depression, indifference, physical changes), and problem resolution (resilience and annual ceremonies). Healthy mourning is crucial. Because mourning significantly contributes to maintaining balance or tranquility in an individual's life. Essentially, mourning helps individuals recover from the impact of major and minor losses experienced in life. Failing to go through the mourning process naturally may lead to psychological and even physical harm in women. In such cases, feelings of worthlessness and negative body image are felt by women. Stronger feelings of guilt, self-blame, anger, fear, sorrow, and grief emerge more intensely in these women. Beliefs, moral values, and the desire for motherhood become conflicted due to experiencing this loss, leading to a psychological turmoil that results in various mental disorders, including depression, anxiety attacks, suicidal thoughts, sexual dysfunction, sleep disturbances, reduced self-esteem, eating disorders, and difficulties in establishing relationships with other children, ultimately affecting the quality of life. On the other hand, women who have experienced unsuccessful pregnancies such as miscarriage and stillbirth are more likely to be at risk of anxiety and reduced quality of life compared to women without this experience. Various studies have described a high prevalence of anxiety and depression symptoms following perinatal childbirth. (10-13)

One of the most effective ways to combat anxiety and depression is through non-pharmacological methods. Studies have shown that educational and skill-building interventions are effective in reducing depression and can improve the quality of life for mothers. (24) Recently, the use of complementary therapies in treating depression has been under scrutiny. One of the newest and most effective complementary methods is art therapy, whose functions in addressing mental health problems have been confirmed. (25) In recent years, art therapy has gained a special position in the field of treatment as a branch of modern medicine. Some branches of this therapy, such as music and painting, have become more popular in our country. Art therapy is defined as "the use of artistic materials to express and empower the patient through recognizing and resolving their inner conflicts in the presence of a trained art therapist." In art therapy, patients do not

need prior skills or experience in art; rather, through creating artistic works and reflecting on the product and process, they increase their self-awareness and positive emotions, enhancing self-confidence. (27) Art therapy requires attention to patients' psychological needs, interpretation, the need for freedom, expression of existence, and tranquility. Therefore, it does not focus on the details of artistic skills but rather on the role of psychotherapeutic and therapeutic approaches in their activities and artistic productions. Art therapy is widely used for the treatment of mental disabilities, learning disorders, academic failures, anxiety, depression, and obsessive-compulsive disorder. The use of painting, sculpting, photography, collage, music, performance, and poetry form various types of art therapy, presented under titles such as music therapy, drama therapy, narrative therapy, and painting therapy. (28) Art therapy raises awareness of suppressed emotions and reveals them through created images. It is utilized in various groups for controlling stress, anxiety, depression, fear, and improving the quality of life, with focus areas in women and maternal health including depression in menopausal women, breast cancer patients, and postpartum depression. (29-33)

Considering that the responsibility of fertility health service providers is to pay attention to all aspects of fertility health, its process, and function, it seems that in the pursuit of this matter, dealing with the issue of grief and considering the consequences of this phenomenon has not received enough attention in assessing health services. This type of care is lacking in both the perspective of service providers and patients. On the other hand, given the international focus on empowering women to take care of themselves, which has been a major focus in recent years, facilitating women's mental well-being is also considered an important goal of maternal care. (9,34) Healthcare professionals play a significant role in creating positive changes in parents to prepare them for pregnancy and healthy parenting in the future. (35)

Therefore, considering the burden of grief after miscarriage and the likelihood of it progressing towards depression, as well as the current interest and inclination of society towards alternative and non-drug therapies, and the limited research in this area, the necessity of conducting such important and practical research seems inevitable

Implementation Method:

Study Type: The present study is a parallel randomized clinical trial involving 60 mothers (30 in the intervention group and 30 in the control group) who attend the hospitals of Tehran University of Medical Sciences following pregnancy loss. Individuals who meet the inclusion criteria, including a history of recent miscarriage or stillbirth within the last 6 weeks, having at least basic literacy skills, willingness to participate in the study, confirmation of grief using the PGS questionnaire, and residing in Tehran, will be enrolled in the study. At the beginning of the visit, the Perinatal Grief Scale (PGS) will be completed by the participants. Those scoring 92 or higher on the total grief score, or 34 or higher on the active grief or 30 or higher on the difficulty coping subscale, or 27 or higher on the despair subscale, indicating high levels of grief, will be included in the study. Randomization Method: This will be carried out using a block randomization method. Four-person blocks are selected so that Group A will be the intervention group and Group B will be the control group. Each of the six possible scenarios will be written on separate cards, numbered from one to six. The numbers will then be written on paper, randomly selected, until the expected number of samples is complete. The concealment of the sequence will be done using sealed envelopes, each containing one of the designated letters in order, sealed, and given to the researcher. The randomization and concealment process will be performed by a statistical consultant. Blinding Procedure: Due to the nature of the intervention, blinding is not feasible. Subsequently, participants will be informed about the study and will complete an informed consent form. Demographic, grief, anxiety, and quality of life questionnaires will be completed by them. The selected samples will undergo four 90-minute sessions of art therapy and pottery weekly for two weeks according to the designed program detailed below, and anxiety and grief questionnaires will be completed after two sessions of art therapy and at the end of the intervention. Eight weeks after the intervention, the questionnaires will be completed again. During the intervention period, the control group will not receive any art therapy interventions, except for routine medical care following pregnancy loss. To ensure research ethics, all participants will be assured of the confidentiality of the research results. Additionally, after the intervention period, a condensed intervention (2 sessions) will be offered to the control group if the individuals are willing. To combat Miss to follow up issues, participants will be reminded of their class attendance one day before each art therapy session through SMS messages. The completion of questionnaires eight weeks after the intervention will be done electronically.

Session Plan for Art Therapy Intervention:

First Session: Warm-up Exercise: Introduction to Colors Main Exercise: Paper Collage or Images - Creating a Family Tree, Depicting Current State Relaxation Exercise: Listening to Instrumental Music and Relaxation Purpose: Familiarity with group members, Self-expression Duration: 90 minutes

Second Session: Warm-up Exercise: Color Spots Main Exercise: Full-length Image, Painting Worst Fears & Threatening Situations, Stress Ballooning Relaxation Exercise: Closing Eyes and Imagining a Calming Place Purpose: Expressing Anger, Fear, Stress Duration: 90 minutes

Third Session: Warm-up Exercise: Contrasting Colors, Lines, and Shapes Main Exercise: Pockets and Boxes - Self-portrait, Symbolic Representation of Talents or Abilities, Drawing a Support Circle and Peaceful Painting Relaxation Exercise: Looking in the Mirror and Showing Self-compassion Purpose: Self-worth, Abilities, Support Duration: 90 minutes

Fourth Session: Warm-up Exercise: Creating Personal Symbol with Clay Main Exercise: Making Seasons with Clay, Creating a Nest Tree and Bird and Storm-affected Tree, Writing a Letter to God and a Letter to Spouse Relaxation Exercise: Breathing and Music, Gratitude Purpose: Insight, Solutions, Hope and Future Duration: 90 minutes

Reducing Implementation Constraints:

Intervention variables beyond control in this research include: mental and psychological status, cultural and social backgrounds, individual differences among patients, and the complexity of the grief issue in different individuals. These factors may impact the research results. Providing assurance to participants regarding the confidentiality of information can largely address this issue, but complete control is beyond the researcher's capabilities.

References:

1)Ekström A and Nissen E (2006) A mothers feelings for her infant are strengthened by excellent breastfeeding counseling and continuity of care. Pediatrics. 118 (2) e309-e14.

.2)Pazandeh F, Sheikhan Z, Sharghie Someah N. Psycological health and woman. ۱st ed. Tehran: Tohfeh Publication 2007; p:70.

3) Davudsdottir, R. (2012). Anthropology of Childbirth Cross-cultural approach. Unpublished BA Thesis, University of island.

4) Goodman P, Mackey M C and Tavakoli A S (2004) Factors related to childbirth satisfaction. Journal of Advanced Nursing. 46 (2) 212-9.

5) M. Tomlinson P. J. Cooper A. Stein L. Swartz C. Molteno Post-partum depression and infant growth in a South African peri-urban settlement, J: child:care, health and development, ۰ January 2006

6)Korja R, Savonlahti E, Haattaja L, et al. Attachment representations in mothers of preterm infants. Infant Behav Dev 2009;32 (3):305-11

7)Soltani F, Shobeiri F. Mensatrual patterns and its disorders in high school girls. The Iraian J of Obstetrics , Gynecology & Infertility. 2011;14(1):28-36. Lau DH, Patient empowerment--a patient-centred approach to improve care. Hong Kong Med J Vol ۸ No ۵ October .2002.

8) Myles textbook for midwives. Translated by Fatemeh Vasegh R. (2015).

9) Janati Y. (2013). Psychiatry in midwifery..

10). Helen Statham. The effects of miscarriage and other ‘unsuccessful’ pregnancies on feelings early in a subsequent pregnancy. Journal of Reproductive and Infant Psychology.Volume 12, 1994- Issue 1.

11)Evelyn Regina Couto, Quality of life, depression and anxiety among pregnant women with previous adverse pregnancy outcomes, Sao Paulo Med. J. vol.۱۲۷ no.۴ São Paulo July 2009.

https://www.who.int/healthinfo/survey/whoqol-qualityoflife/en/2019

13). Adeyemi A, Mosaku K, Ajenifuja O, Fatoye F, Makinde N, Ola B. Depressive symptoms in a sample of women following perinatal loss. J Natl Med Assoc. 2008,100(12):1463-8.

14.F.Gary Cunningham. Williams Obstetrics. 25th edition2018..

15)Susheela Singh, Abortion Worldwide 2017: Uneven Progress and Unequal Access.2017.

16)Abortion Worldwide 2017: Uneven Progress and Unequal Access. SusheelaSingh,LisaRemez,Gilda,Sedgh,Lorraine,KwokandTsuyosh.https://www.who.int/maternal_child_adolescent/epidemiology/stillbirth/en/2018.
17)Badenhorst W, Hughes P. Psychological aspects of perinatal loss. Best Practice and Research in Clinical Obstetrics and Gynaecology. 2007;21,:249-259

18) van der Sijpt E. Marginal matters: Pregnancy loss as a social event. Social Science & Medicine. 2010;71(10):1773-79
19)Bloom SS, Wypij D, Gupta MD. Dimensions of womens autonomy and the influence on maternal health care utilization in a North Indian City. Demography. 2001;38(1):67–78.

20). Frost M, Condon JT. The psychological sequelae of miscarriage: a critical review of the literature. Austr N Z J Psychiatry. 1996

21)Janssen HJ, Cuisinier MC, de Graauw KP, Hoogduin KA. A prospective study of risk factors predicting grief intensity following pregnancy loss. Arch Gen Psychiatry. 1997;54(1):56-61. doi: ۱۰.۱۰۰۱/archpsyc.
22). Saflund K, Wredling R. Differences within couples' experience of their hospital care and well-being three months after experiencing a stillbirth. Acta Obstetrica et Gynecologica Scandinavica. 2006;85:
23). Hassan Zahraee R. Fahami F. Yazdani M. Ahmadi Z. Bashardoost N. supportive role of the midwife in preventing postpartum depression, J: Qazvin Univ. of Med. Sci. No, 25, spring 2003

24) Perry C۱, Thurston M, Osborn T. Time for Me: the arts as therapy in postnatal depression. Complement Ther Clin Pract. 2008 Feb;14(1):38-45. doi: ۱۰.۱۰۱۶/j.ctcp.۲۰۰۷.۰۶.۰۰۱.
25)Rouhe H, Salmela-Aro K, Toivanen R, Tokola M, Halmesmäki E, Ryding E-L, et al. Group psychoeducation with relaxation for severe fear of childbirth improves maternal adjustment and childbirth experience-a randomised controlled trial. Journal of Psychosomatic Obstetrics & Gynecology. 2014.
26)Mohammedian Y, Shahidi Sh, Zadeh Mohammadi A, Mahaki B, Evaluating the use of poetry

to reduce signe of depression in students. J: Scientific Journal of Ilam university of medical sciences. 2009

27). Safran, D.S. (2002).Art therapy and AD/HD:Diagnostic and therapeutic approach. London: Jessica Kingsley.

28). Afnan Hamed-Agbariah, Rosenfeld Y.The added value of art therapy for mothers with post partum depression in Arabic society in Israel. Harefuah. 2015 Sep;154(9):568-72.

29). Svensk AC١, Oster I, Art therapy improves experienced quality of life among women undergoing treatment for breast cancer: a randomized controlled study. Eur J Cancer Care (Engl). 2009 Jan;18(1):69-77. doi: ١٠.١١١١/j.١٣۶۵-٢٣۵۴.٢٠٠٨.٠٠٩۵٢.x.

30)Eliana C. Ciasca,١ Rita C. Ferreira. Art therapy as an adjuvant treatment for depression in elderly women: a randomized controlled trial. Revista Brasileira de Psiquiatria. 2018,Brazilian Journal of Psychiatry.

31). Wahlbeck H, Kvist LJ, Landgren K. Gaining hope and self-confidence-An interview study of womens experience of treatment by art therapy for severe fear of childbirth. Women Birth. 2018 Aug;31(4):299-306. doi: ١٠.١٠١۶/j.wombi.٢٠١٧.١٠.٠٠٨.

32). Lau DH, Patient empowerment--a patient-centred approach to improve care. Hong Kong Med J Vol 8 No 5 October ٢٠٠٢.

33). Tonia M. Cassaday, Impact of Pregnancy Loss on Psychological Functioning and Grief Outcomes, obstetrics and gynecology clinics, September 2018Volume 45, Issue 3, Pages 525-33

34). Armstrong DS. Emotional distress and prenatal attachment in pregnancy after perinatal loss. J Nurs Scholarsh. 2002;34(4):339-45

35) Dwight L. Evans, Edna B, Treating and Preventing Adolescent Mental Health Disorders: What We Know and What We Dont Know. A Research Agenda for Improving the Mental Health of Our Youth, Oxford University Press, Aug 2012..
